# The interaction between dietary nitrates/nitrites intake and gut microbial metabolites on metabolic syndrome: a cross-sectional study

**DOI:** 10.3389/fpubh.2024.1398460

**Published:** 2024-09-11

**Authors:** Atieh Mirzababaei, Maryam Mahmoodi, Abbasali Keshtkar, Sara Ebrahimi, Fereshteh Pashayee-Khamene, Faezeh Abaj, Mina Radmehr, Pardis Khalili, Mahya Mehri Hajmir, Khadijeh Mirzaei

**Affiliations:** ^1^Department of Community Nutrition, School of Nutritional Sciences and Dietetics, Tehran University of Medical Sciences, Tehran, Iran; ^2^Department of Cellular and Molecular Nutrition, School of Nutritional Science and Dietetics, Tehran University of Medical Sciences, Tehran, Iran; ^3^Department of Disaster and Emergency Health, School of Public Health, Tehran University of Medical Sciences, Tehran, Iran; ^4^Institute for Physical Activity and Nutrition (IPAN), School of Exercise and Nutrition Sciences, Deakin University, Geelong, VIC, Australia; ^5^Student Research Committee, Ahvaz Jundishapur University of Medical Sciences, Ahvaz, Iran; ^6^Department of Nutrition, Dietetics and Food, Monash University, Clayton, VIC, Australia; ^7^Department of Nutrition, Science and Research Branch, Islamic Azad University, Tehran, Iran; ^8^Department of Exercise and Nutrition Sciences, Milken Institute School of Public Health, The George Washington University, Washington, DC, United States

**Keywords:** nitrates, nitrites, gut microbial metabolites, interaction, metabolic syndrome, KYN, TMAO

## Abstract

**Background:**

Metabolic syndrome (MetS) prevalence has increased globally.The evidence shows thatdiet and gut microbial metabolites includingtrimethylamine N-oxide (TMAO) and kynurenine (KYN) play an important role in developing MetS. However, there is a lack of evidence on associations between between diet and these metabolites. This study aimed to investigate the interaction between dietary nitrate/nitrite and gut microbial metabolites (TMAO, KYN) on MetS and its components.

**Methods:**

This cross-sectional study included 250 adults aged 20–50 years. Dietary intake was assessed using food frequency questionnaires (FFQ), and serum TMAO and KYN levels were measured. MetS was defined usingthe National Cholesterol Education Program Adult Treatment Panel (NCEP ATP III) criteria.

**Result:**

The ATPIII index revealed an 11% prevalence of metabolic syndrome among the study participants. After adjusting for confounders, significant positive interactions were found: High animal-source nitrate intake and high TMAO levels with elevated triglycerides (TG) (p interaction = 0.07) and abdominal obesity (p interaction = 0.08). High animal-source nitrate intake and high KYN levels with increased TG (p interaction = 0.01) and decreased high-density lipoprotein cholesterol (HDL) (p interaction = 0.01).Individuals with high animal-source nitrite intake and high TMAO levels showed increased risk of hypertriglyceridemia (OR: 1.57, 95%CI: 0.35–2.87, *p* = 0.05), hypertension (OR: 1.53, 95%CI: 0.33–2.58, *p* = 0.06), and lower HDL (OR: 1.96, 95%CI: 0.42–2.03, *p* = 0.04). Similarly, high animal-source nitrite intake with high KYN levels showed lower HDL (OR: 2.44, 95%CI: 1.92–3.89, *p* = 0.07) and increased risk of hypertension (OR: 2.17,95%CI: 1.69–3.40, *p* = 0.05). Conversely, Negative interactions were found between high plant-source nitrate/nitrite intake with high KYN and TMAO levels on MetS and some components.

**Conclusion:**

There is an interaction between dietary nitrate/nitrite source (animal vs. plant) and gut microbial metabolites (TMAO and KYN) on the risk of of MetS and its components. These findings highlight the importance of considering diet, gut microbiome metabolites, and their interactions in MetS risk assessment.

## Introduction

The prevalence of MetS has increased worldwide (1). Approximately a quarter of the world’s population experienced MetS in 2018 (2). According to a study in 2020, the prevalence of metabolic syndrome increased from 5.2 to 7.3% from between 2009 and 2017 (3). The MetS is diagnosed based on developing abdominal obesity, low levels of HDL-C, dyslipidemia including high triglycerides and low HDL-C, insulin resistance, and hypertension (4). Based on a recent population-based study in 2021, the prevalence of MetS in the Iranian adult population was around 47% (5). Various factors, including genetics (6), physical activity (PA) (7), smoking (8), diet (9), and Gut Microbial Metabolites (10) are associated with the incidence of MetS. Diet plays an important role in the occurrence or prevention of MetS (11). Nitrate and nitrite are found in fruits and vegetables, drinking water, and processed foods (12). Dietary nitrate benefits MetS, through providing antioxidant, anticoagulant and anti-inflammatory effects and blood pressure reduction (13–15). Furthermore, dietary nitrate can be used therapeutically against obesity and related metabolic complications by increasing the mitochondrial respiration of fat and reducing oxidative stress and inflammation (15).

Diet can change the production pathway of gut metabolites. A study reported that the nitrate in intestines could increase the production of Trimethylamine (TMA) by *E. coli* bacteria (16). Our recent study found positive associations between dietary nitrate and nitrite intake from animal sources and gut microbial metabolites KYN and TMAO (17). In addition, a recent animal study reported that a combination of dietary nitrates and gut microbiota could reduce serum lipids and glucose intolerance (18, 19). This evidence shows the importance of the nitrate-intestine-MetS axis and the potential role of the gut microbiome in the development of MetS (18–20). It is worth noting that animal-based foods includingbeef, pork, lamb, and veal, contain substances that can be converted into TMAO in the body. When these foods are consumed, gut microbes use dietary precursors includingcholine, L-carnitine, lecithin, phosphatidylcholine, and betaine to produce trimethylamine (TMA), releasing TMA into the bloodstream, which is converted to TMAO by the liver enzyme flavin-containing monooxygenase-3 (FMO3) (21, 22). Flavin-containing monooxygenase 3 (FMO3), the enzyme responsible for TMAO production is involved in obesity and the beginning of white adipose tissue. Furthermore, elevated TMAO levels may increase hepatic insulin resistance, leading to obesity (23, 24).

TMAO, derived from choline and L-arginine, can increase platelet accumulation, which results in atherosclerosis and higher blood pressure (25). TMAO exerts its effect on cardiovascular diseases (CVD) by influencing the expression of macrophage low-density lipoprotein cholesterol (LDL) receptors, changing the phenotype of endothelial cells and macrophages, stimulating platelet hyperreactivity, and increasing lipid-laden macrophages (26, 27). Furthermore, the previousstudies demonstrated the protective role of TMAO against MetS and its components (25, 28). There are several possible ways that dietary nitrates and nitrates could affect KYN levels. KYN production may be impacted by nitrate and nitrite through their conversion to Nitric Oxide (NO).^1^ Indoleamine 2,3-dioxygenases (IDO) are tryptophan-degrading enzymes regulated by NO which are involved in the metabolism of tryptophan and the synthesis of KYN (29). Furthermore, KYN levels are positively associated with higher Homeostatic Model Assessment-Insulin Resistance (HOMA-IR) and body mass index (BMI) (30). Inflammation increases KYN levels, which has a positive association with TG, BMI, LDL, and WC while having an inverse relationship with HDL (31). Our recent research showed a positive association between serum TMAO and KYN levels and MetS and some of its components (32). Also, Numerous chronic inflammatory metabolic disorders, includingMets and atherosclerosis, are known to be risk factors for CVD and Kynurenine pathway (KP) may play a role in their development (33–35). Patients with atherosclerotic lesions and ischemic heart disease have been found to have higher levels of tryptophan (TRP)^2^ degradation via KP (34). Furthermore, it has been demonstrated that overweight and dyslipidemia are positively connected with elevated IDO activity and KYN (34, 36). Studies on the association between dyslipidemia and the KYN pathway, however, are still conflicting and unclear. An experimental study conducted on IDO-deficient mice found an increase in TG levels and a decrease in HDL levelsin (37). However, an animal study found that 3-hydroxy anthranilic acid, one of the KP’s downstream metabolites, improved atherosclerosis in LDLR−/−mice by reducing the accumulation of hepatic fat and lowering plasma levels of cholesterol and triglycerides (TG) (38).

However, the current evidence on the role of KYN in MetS development is mixed. Further studies are needed to clarify the existing associations (33, 36, 39, 40).

While the evidence shows the association between dietary nitrate and nitrite intake and gut microbiota metabolites and MetS components (18, 19), no previous study has investigated the role of combining effects of dietary nitrate and nitrite intake and gut microbial metabolites KYN and TMAO on MetS. As a result, this study for the first time, aimed to assess the interaction between dietary nitrate and nitrite intake and gut microbial metabolites KYN and TMAO on MetS and its components.

## Methods

### Study design and population

This cross-sectional study was performed on 250 individuals aged between 20 and 50 in the baseline phase of the Tehran University of Medical Sciences (TUMS) Employee’s Cohort study (TEC) between 2018 and 2020. This study was conducted according to the guidelines in the Declaration of Helsinki. The ethics committee of the TUMS (IR.TUMS.MEDICINE.REC.1401.064) approved the protocol of the present study. All participants signed the consent form before entering the study (41). Participants with a disease history including diabetes mellitus, cancers, hepatic, kidney disease, and polycystic ovary syndrome (PCOS) were not eligible. Participants without MetS were excluded. Further exclusion criteria were pregnancy and lactation, alcohol consumption, and adherence to particular dietary intake. Furthermore, participants who took medications that affect glucose and/or lipid-lowering and/or body weight were notincluded in this study. Also, participants with arbitrary dietary patterns or weight fluctuations of 5% over the past year and whose energy intake was less than 800 or more than 4,200 (kcal/day) were excluded.

### Dietary intake measurement

Participants’ dietary intake was evaluated using a valid and reliable 144-item FFQ. The questionnaire contained mixed dishes (cooked or canned), grains (different types of bread, cakes, biscuits, and potato), dairy products (dairy, butter, and cream), fruits, vegetables, miscellaneous food items and beverages (including sweets, fast foods, nuts, desserts, and beverages). Common portion sizes were used to determine the quantity of foods consumed. Nine multiple-choice frequency response categories varying from “never or less than once a month” to “6 or more times per day” were administered to report participants’ dietary intakes. In this regard, the National Nutrient Data Bank of the United States Department of Agriculture (USDA) was used to assess daily food intake, and the NUTRITIONIST 4 (First Data Bank, San Bruno, CA) food Analyzer was used to analyze nutrient intake (42).

### The measurement of nitrate and nitrite intake

Given the limitations of data on the nutrient content of beverages and raw foods in the Iranian food composition table, the food composition table of the USDA (except for nitrates and nitrites) was used (29). A recent Iranian survey on the frequent food items consumed estimated the level of nitrate and nitrite and their food composition values. Nitrate and nitrite content of food groups were estimated based on a previous Iranian study (43).

### Biochemical indicators assessment

To evaluate biochemical tests, venous blood from all individuals after 12–14 h of overnight fasting hours was taken. The blood samples were centrifuged for about 10 min at 3000 rpm. The biochemical analyses were conducted using commercial kits (Pars Azmoon Inc., Tehran, Iran). Total cholesterol (TC), TG, LDL-C, HDL-C, and fasting blood sugar (FBS) were evaluated using an enzymatic colorimetric method and phosphor tungstic acid. Enzymatic methods were used to measure serum concentrations of alkaline phosphatase (ALK), aspartate aminotransferase (AST), alanine aminotransferase (ALT), KYN, and TMAO. Analyses were carried out using commercial kits (Shanghai Crystal Day Biotech, Shanghai, China).

### Blood pressure and heartbeat assessment

To measure systolic blood pressure (SBP), diastolic blood pressure (DBP), and heartbeat, a standardized mercury sphygmomanometer was used three times in a sitting position, at time intervals of the first 20 min, then 2 h, and 4 h after admission. The average of these three measurements was recorded.

### Definition of MetS and its components

According to the diagnostic criteria proposed by the National Cholesterol Education Program Adult Treatment Panel III (NCEP ATP III), MetS was characterized as having at least 3 of the metabolic criteria: (1) Hyperglycemia as FBS ≥100 mg/dL (5.6 mmol/L), (2) Hypertriglyceridemia as serum TG ≥150 mg/dL (1.69 mmol/L), (3) Low HDL-C as serum HDL-C < 40 mg/dL (1.03 mmoL/L) in men and < 50 mg/dL (1.29 mmoL/L) in women, (4) Hypertension as BP ≥130/85 mmHg, and (5) Abdominal obesity as WC >90 cm in men and > 80 cm in women. However, based on NCEP ATP III (44, 45), modified for the Iranian population, WC >95 cm was considered abdominal obesity.

### Assessment of anthropometric indices

The height was measured without shoes and in a standing normal position using a non-elastic measuring tape with about 0.1 cm precision. Participants’ WC was measured using a non-elastic tape measure with about 0.1 cm accuracy from the narrowest area between the lower rib margin and iliac crest. Hip circumference (HC) was assessed using an inelastic tape measure with 0.1 cm accuracy at the widest part of the buttocks. The Waist-hip ratio (WHR) was calculated by dividing WC by HC. BMI (weight/height (kg/m^2^)) was calculated for every participant (46).

### Assessment of other variables

The validated and reliable self-report of the International Physical Activity Questionnaire (IPAQ) was used to assess PA. This questionnaire has previously been utilized in Iranian adults (47). The metabolic equivalent (MET) was recorded by observing the PA level in the last 7 days (48). Scores were calculated according to the frequency and time spent on light, moderate, high, and very high-intensity activities. The scores for different activities were summed up to obtain the total MET-min/week. The education, job, marital status, smoking, socioeconomic status (SES), medications, and supplementation intake were assessed using a self-report socio-demographic questionnaire.

### Statistical analysis

The Kolmogorov–Smirnov test was utilized to assess the normality of distribution. The data were presented as numbers (%) for categorical variables and mean ± SD for continuous variables. The chi-square test was used to compare the categorical variables. Cutoffs of TAMO and KYN were defined based on the median. The Comparison between nitrate and nitrite intake and TMAO and KYN levels was performed using independent samples *T*-tests and analysis of covariance (ANCOVA), adjusted for age, energy intake, physical activity, sex, and BMI. A binary Logistic regression analysis was used to assess the interaction between the median of nitrate, nitrite intake, and gut metabolites on continuous variables. The analysis was adjusted for age, energy intake, BMI, PA, smoking, sex, education, marriage status, SES, use of multivitamins,and intake of vitamin C. SPSS (version 25; SPSS Inc., IL) and STATA (version 17) was used to perform the statistical analysis. *p* < 0.5 was considered statistically significant, while *p* < 0.01 was statistically significant for interactions.

## Results

### Study population characteristics

This cross-sectional study was conducted on 250 Iranian men and women. The mean age, weight, height, and WC of participants were 40.96 (8.66) years, 74.12 (14.47) kg, 164.73 (8.82) cm, and 90.63 (11.53) cm, respectively. The majority of participants were female (73%) and the prevalence of metabolic syndrome was found to be approximately 11% in the studied subjects based on the ATPIII index. The median and interquartile range (IQR) of total nitrate and nitrite intakes were 631.69 (509.92–772.58) and 12.18 (9.79–14.87) mg/day. The minimum and maximum levels for KYN were 55.30 and 1782.85 nmol/L, respectively, while they were 1.80 and 242.99 pg./mL for TMAO.

### Characteristics of study participants according to the median of TMAO and KYN

Characteristics of participants according to TMAO and KYN median are presented in Tables 1, 2. Participants were stratified into two groups based on the median of TMAO (30.39 pg./mL) and KYN (297.18 nmol/L). In both crude and adjusted models, individuals with higher TMAO levels had higher mean WC, HC, TG, FBS, and ALT. After adjusting for confounders (age, energy intake, PA, sex, and BMI), participants with higher TMAO levels had higher WHR and AST.

**Table 1:** Characteristics of participants based on TMAO and KYN levels.

	TMAO		*p* value	*P* value *	KYN		*P* value	*P* value *
Variables	<30.39 pg/mL *n* = 124	≥30.39 pg/mL *n* = 126			<297.18 nmoL/L *n* = 124	≥297.18 nmoL/L *n* = 126		
Demographic								
Age (year)	40.99 (9.18)	40.93 (8.16)	0.95	0.66	40.92 (8.83)	40.99 (8.52)	0.37	0.56
Sex								
Male	39 (58.2)	28 (41.8)	0.09	**0.06**	36 (52.2)	33 (47.8)	0.66	0.52
Female	85 (46.4)	98 (53.6)			88(49.2)	93 (50.8)		
Anthropometric								
Weight (kg)	75.69 (14.72)	78.16 (14.72)	0.19	0.21	75.75 (15.10)	78.12 (15.16)	0.21	0.10
Height (cm)	166.01 (8.94)	166.10 (9.41)	0.58	0.88	165.70 (8.89)	166.41 (9.45)	0.15	0.59
BMI (kg/m^2^)	26.99 (4.40)	27.48 (4.56)	0.40	0.57	27.05 (4.72)	27.41 (4.25)	0.44	0.71
WHR	0.85 (0.08)	0.89 (0.07)	0.30	**0.01**	0.87 (0.08)	0.94 (0.09)	0.41	**0.05**
WC (cm)	89.34 (12.08)	91.91 (10.85)	**0.05**	**0.01**	89.91 (11.13)	96.36 (11.91)	**0.03**	**<0.001**
HC (cm)	103.68 (8.61)	105.49 (7.89)	**0.04**	**0.007**	103.51 (8.08)	109.67 (8.39)	**0.03**	**0.002**
Blood parameters								
TG (mg/dl)	142.37 (68.42)	169.87 (115.34)	**0.02**	**0.001**	164.88 (112.30)	147.60 (75.32)	0.14	0.24
HDL (mg/dl)	44.35 (8.27)	42.68 (8.33)	0.11	0.23	44.08 (8.49)	42.93 (8.15)	0.27	0.43
LDL (mg/dl)	103.69 (28.08)	107.41 (26.35)	0.28	0.27	104.09 (27.64)	106.97 (26.87)	0.40	0.24
TC (mg/dl)	181.16 (36.18)	184.19 (44.67)	0.43	0.24	183.80 (36.76)	187.56 (44.26)	0.51	**0.05**
FBS (mg/dl)	91.28 (25.32)	98.07 (42.66)	**0.04**	**0.02**	95.36 (36.01)	100.05 (34.59)	0.13	**0.04**
ALT (IU/L)	28.94 (19.78)	33.52 (28.94)	**0.02**	**0.001**	27.60 (21.23)	34.80 (27.57)	**0.02**	**0.001**
AST (IU/L)	21.42 (14.01)	24.06 (11.21)	0.39	**0.001**	20.84 (13.76)	22.64 (11.47)	0.26	**0.02**
ALK (IU/L)	172.56 (44.49)	175.10 (48.90)	0.32	0.57	167.84 (43.36)	179.84 (49.23)	**0.04**	**0.03**
Blood pressure								
SBP (mmHg)	115.48 (16.41)	115.73 (15.77)	0.30	0.31	116.28 (15.66)	114.92 (16.49)	0.23	0.82
DBP (mmHg)	77.64 (10.37)	76.32 (9.83)	0.77	0.58	77.74 (10.38)	76.21 (9.79)	0.13	0.64
Categorical variables								
Marriage status								
Married	101 (49.5)	100 (50.5)	0.65	0.73	104 (52.5)	97 (47.5)	0.14	0.12
Single	23 (46.9)	26 (53.1)			20 (40.8)	29 (59.2)		
Education levels								
Under diploma	0 (0)	4(100)	0.54	0.61	0 (0)	3 (100)	0.60	0.78
Diploma	42 (48.8)	44 (51.2)			43 (50)	43 (50)		
University	82 (51.9)	78 (48.1)			81 (50.6)	80 (49.4)		
Job categories								
Office	65 (51.2)	62 (48.8)	0.12	0.24	58 (45.7)	69 (54.3)	**0.03**	**0.02**
Clinical	33 (49.3)	34 (50.7)			39 (58.2)	28 (41.8)		
Technical	9 (31.1)	20 (68.9)			15 (43)	20 (57)		
Security	11 (52.4)	10 (47.6)			12 (57.1)	9 (42.9)		
Socio-economic status								
Low	5 (50)	5 (50)	0.52	0.10	5 (62.5)	3 (37.5)	0.98	0.33
Modrate	110 (48.7)	116 (51.3)			115 (50.2)	114 (49.8)		
High	9 (64.3)	5 (35.7)			4 (30.7)	9 (69.3)		
Physical activity								
Low	120 (50)	120 (50)	0.71	0.45	122 (50.6)	119 (49.4)	0.30	0.41
Modrate	4 (44.4)	6 (55.6)			2 (33.3)	7 (66.7)		
Smoking								
Yes	58 (54.2)	49 (45.8)	0.20	0.56	43 (40.2)	64 (59.8)	**0.007**	**0.008**
No	66 (44.2)	77 (53.8)			81 (57.3)	62 (42.7)		
Supplement Use								
Yes	15 (51.7)	14 (48.3)	0.80	0.72	14 (51.7)	15 (48.3)	0.84	0.97
No	109 (49.3)	112 (50.7)			110 (49.8)	111 (50.2)		

BMI: body mass index, WHR: waist to hip ratio, WC: waist circumference, HC: hip circumference, TG: triglyceride, HDL: high-density lipoprotein, LDL: low-density lipoprotein, TC: total cholesterol, FBS: fasting blood sugar, ALT: alanine transaminase, AST: aspartate aminotransferase, ALK: alkaline Phosphatase, SBP: systolic blood pressure, DBP: diastolic blood pressure, TMAO: trimethylamine N-Oxide, KYN: kynurenin. Continuous variables were presented as means (± SD) obtained from the independent *t*-test. Categorical variables were presented as N (%) obtained from the chi-square analysis. *p*-values < 0.05 were considered significant. *p*-value*: the analysis was adjusted for age, energy intake, physical activity, sex, and BMI.

**Table 2:** Dietary intake of participants based on TMAO and KYN levels.

	TMAO		*P* value	*P* value *	KYN		*P* value	*P* value *
Variables	<30.39 pg/mL *n* = 124	≥30.39 pg/mL *n* = 126			<297.18 nmoL/L *n* = 124	≥297.18 nmoL/L *n* = 126		
Kcal (kcal/day)	2717.43 (731.07)	2567.53 (657.71)	0.12	0.13	2686.67 (698.77)	2595.54 (698.14)	0.35	0.34
Cho (gr/day)	385.28 (103.80)	362.95 (104.41)	0.13	0.97	378.55 (99.79)	369.51 (109.74)	0.54	0.42
Fat (gr/day)	88.03 (32.69)	84.22 (25.44)	0.35	0.71	88.57 (31.89)	83.45 (26.09)	0.21	0.29
Protein (gr/day)	104.83 (30.85)	97.54 (26.49)	0.07	0.27	102.74 (29.92)	99.57 (27.88)	0.44	0.94
Nitrate								
Total (mg/day)	699.20 (241.99)	645.54 (241.04)	0.11	0.41	679.77 (260.32)	664.95 (221.84)	0.66	0.85
Animal source (mg/day)	25.26 (11.95)	22.90 (8.42)	0.10	0.59	24.57 (11.27)	23.56 (9.37)	0.49	0.71
Plant source (mg/day)	648.92 (225.67)	610.71 (232.73)	0.23	0.63	632.75 (236.33)	627.14 (222.61)	0.86	0.41
Nitrite								
Total (mg/day)	12.98 (4.29)	12.16 (3.54)	0.14	0.51	12.85 (4.37)	12.26 (3.41)	0.28	0.50
Animal source (mg/day)	7.11 (3.34)	6.35 (2.46)	**0.06**	0.98	7.01 (3.29)	6.44 (2.51)	0.17	0.26
Plant source (mg/day)	5.82 (1.69)	5.78 (2.06)	0.90	0.68	5.81 (1.92)	5.79 (1.84)	0.96	0.52

CHO: cholostreol, TMAO: trimethylamine N-Oxide, KYN: kynurenin. Continuous variables were presented as means (± SD) obtained from the independent *t*-test. *P*-values < 0.05 were considered significant. *P*-value*: the analysis was adjusted for age, energy intake, physical activity, sex, and BMI.

Furthermore, in both crude and adjusted models, participants with higher levels of KYN had higher mean WC, HC, ALT, and ALK. After adjusting for confounding factors, participants with higher levels of KYN had higher mean WHR, TC, FBS, and AST(*p* < 0.05).

### Characteristics of participants according to nitrate intake from animal and plant sources

Table 3 shows the characteristics of participants based on high/low nitrate intake and its dietary source (animal or plant). After adjusting for confounders, participants with higher nitrate intake (≥631.25 mg/day) had lower FBS, ALT, AST, and SBP levels. Furthermore, participants with higher nitrate intake from animal sources (≥22.28 mg/day) had higher LDL, FBS, and SBP levels. On the other hand, participants with higher intakes of nitrate from plant sources (≥595.18 mg/day) had lower BMI, WC, TG, FBS, ALT, AST, and SBP levels.

**Table 3:** Characteristics of the participants among nitrite intake.

	Total		*P* value	*P* value *	Animal source		*P* value	*P* value *	Plant source		*P* value	*P* value *
Variables	>12.15 mg/day *n* = 113	≥12.15 mg/day *n* = 137			<6.27 mg/day *n* = 112	≥6.27 mg/day *n* = 138			<5.62 mg/day *n* = 109	≥5.62 mg/day *n* = 141		
Demographic												
Age (year)	41.46 (8.71)	41.25 (9.15)	0.49	0.41	41.69 (8.77)	41.04 (9.04)	**0.04**	**0.05**	41.37 (8.65)	41.36 (9.15)	0.98	0.40
Sex												
Male	31 (30)	70 (70)	0.56	0.42	33 (32.6)	68 (67.4)	0.91	0.13	34 (33.6)	67 (66.4)	0.96	0.78
Female	82 (55)	67 (45)			79 (53)	70 (47)			75 (50.3)	74 (49.7)		
Anthropometric												
Weight (kg)	73.82 (14.40)	73.77 (15.33)	0.92	0.49	73.63 (14.45)	75.96 (15.17)	0.34	**0.04**	73.86 (14.59)	73.73 (15.04)	0.80	0.48
Height (cm)	164.80 (9.30)	165.04 (9.23)	0.45	0.86	164.72 (9.18)	165.09 (9.35)	0.27	0.46	164.73 (9.36)	165.08 (9.17)	0.29	0.38
BMI (kg/m^2^)	26.90 (5.14)	26.54 (4.28)	**0.04**	**0.01**	26.59 (4.60)	27.89 (4.96)	0.08	**0.05**	26.63 (4.69)	26.85 (4.87)	0.21	0.11
WHR	0.84 (0.083)	0.843 (0.08)	0.75	0.83	0.84 (0.08)	0.84 (0.08)	0.59	0.99	0.84 (0.08)	0.84 (0.08)	0.35	0.61
WC (cm)	87.37 (11.34)	87.23 (11.97)	0.72	0.93	87.25 (11.79)	88.37 (11.45)	0.17	**0.05**	87.54 (11.47)	87.08 (11.77)	0.27	0.18
HC (cm)	103.46 (8.35)	103.34 (8.05)	0.68	0.68	103.40 (8.44)	103.41 (7.99)	0.98	0.93	103.52 (8.26)	103.29 (8.17)	0.44	0.35
Blood parameters												
TG (mg/dl)	123.78 (69.25)	121.99 (74.47)	0.76	**0.06**	123.49 (75.96)	123.19 (68.28)	0.90	0.69	123.54 (71.94)	121.15 (72.49)	0.27	**0.04**
HDL (mg/dl)	47.05 (10.02)	46.75 (10.13)	0.40	0.78	47.04 (10.10)	46.79 (10.04)	0.49	0.89	46.85 (9.73)	48.99 (10.40)	0.39	**0.02**
LDL (mg/dl)	104.002 (25.89)	102.57 (26.52)	0.13	**0.04**	103.10 (25.98)	103.63 (26.37)	0.57	0.17	103.76 (25.75)	101.98 (26.59)	0.40	**0.06**
TC (mg/dl)	179.63 (38.19)	178.70 (38.86)	0.50	0.83	178.72 (37.17)	179.72 (39.76)	0.46	0.21	179.45 (37.55)	178.99 (39.40)	0.74	0.69
FBS (mg/dl)	86.05 (20.97)	85.86 (20.24)	0.80	0.72	85.65 (18.62)	87.24 (21.46)	0.42	**0.03**	86.26 (22.50)	85.63 (18.42)	0.39	0.34
ALT (IU/L)	25.90 (19.74)	26.80 (21.52)	0.23	0.15	26.02 (20.07)	26.59 (21.01)	0.44	0.23	26.94 (22.31)	25.65 (18.57)	0.08	**0.009**
AST (IU/L)	19.52 (9.75)	19.14 (10.69)	0.29	0.44	19.17 (10.44)	20.44 (10.13)	0.45	**0.05**	19.80 (11.72)	18.82 (8.59)	**0.008**	**0.005**
ALK (IU/L)	172.81 (48.22)	172.28 (50.69)	0.76	0.21	172.29 (50.57)	172.86 (48.04)	0.74	0.82	173.15 (50.85)	172.00 (47.73)	0.51	0.88
Blood pressure												
SBP (mmHg)	113.87 (13.52)	112.08 (13.62)	**0.07**	**0.05**	112.93 (13.38)	114.11 (13.73)	**0.01**	**0.02**	113.89 (13.83)	110.16 (13.29)	0.13	**0.04**
DBP (mmHg)	75.63 (8.70)	75.07 (8.44)	0.10	0.12	75.08 (8.37)	75.68 (8.79)	**0.05**	0.12	75.50 (8.68)	74.27 (8.50)	0.45	0.13
Pulse	77.58 (8.55)	77.30 (8.66)	0.37	0.40	77.76 (8.54)	77.15 (8.64)	**0.04**	**0.04**	77.43 (8.50)	77.49 (8.70)	0.83	0.60
Categorical variables												
Marriage status												
Married	92 (55.1)	75 (44.9)	0.56	0.54	82 (49.1)	85 (50.9)	0.80	0.79	82 (49.1)	85 (50.9)	0.56	0.69
Single	21 (25.3)	62 (74.7)			30 (36.1)	53 (63.9)			27 (32.5)	56 (67.5)		
Education levels												
Under than diploma	2 (18)	9 (82)	0.53	0.67	13 (65)	7 (35)	0.39	0.32	9 (42.8)	12 (57.2)	0.47	0.65
Diploma	34 (40)	51 (60)			29 (38.7)	46 (61.3)			30 (40)	45 (60)		
University education	77 (50)	77 (50)			69 (48)	75 (52)			69 (45)	84 (55)		
Job categories												
Office	64 (38)	104 (62)	0.42	0.24	56 (47.1)	63 (52.9)	0.22	0.19	54 (45.4)	65 (54.6)	0.20	0.25
Clinical	35 (63.6)	20 (36.4)			31 (56.4)	24 (43.6)			31 (56.4)	24 (43.6)		
Technical	2 (33.3)	4 (66.7)			12 (22.2)	42 (77.8)			14 (25)	42 (75)		
Security	12 (57.1)	9 (42.9)			12 (57.1)	9 (42.9)			11 (52.4)	10 (47.6)		
Socio-economic status												
Low	5 (28)	13 (72)	0.26	0.43	5 (62.5)	3 (37.5)	0.50	0.43	4 (50)	4 (50)	0.96	0.1
Modrate	98 (47)	111 (53)			87 (48.3)	98 (51.7)			90 (50)	90 (50)		
High	10 (43.4)	13 (56.6)			20 (36)	37 (64)			16 (25.4)	47 (74.6)		
Physical activity												
Low	110 (45.2)	133(54.8)	0.46	0.54	98 (50.5)	96 (49.5)	0.25	0.12	98 (50.5)	96 (49.5)	0.69	0.53
Modrate	3 (42.9)	4 (57.1)			14 (25)	42 (75)			12 (21.1)	45 (78.9)		
Smoking												
Yes	45 (55.6)	36 (44.4)	0.87	**0.01**	46 (35.3)	84 (64.7)	0.54	**0.04**	39 (48.1)	42 (51.9)	0.62	0.86
No	68 (40.3)	101 (59.7)			66 (55)	54 (45)			70 (41.5)	99 (58.5)		
Supplement use												
Yes	23 (79.3)	6 (20.7)	**0.007**	0.37	22 (28.3)	56 (71.7)	**0.06**	0.37	24 (30.8)	54 (69.2)	0.56	0.86
No	90 (40)	131 (60)			87 (50.6)	85 (49.4)			85 (49.4)	87 (50.6)		

BMI: body mass index, WHR: waist to hip ratio, WC: waist circumference, HC: hip circumference, TG: triglyceride, HDL: high-density lipoprotein, LDL: low-density lipoprotein, TC: total cholesterol, FBS: fasting blood sugar, ALT: alanine transaminase, ASP: aspartate aminotransferase, ALK: alkaline Phosphatase, SBP: systolic blood pressure, DBP: diastolic blood pressure, TMAO: trimethylamine N-Oxide, KYN: kynurenin. Plant sources include: grains, vegetables, legumes, and nuts. Animal sources include: meat, poultry, processed meats, and dairy products. Continuous variables were presented as means (± SD) obtained from the independent t-test. Categorical variables were presented as N (%) obtained from the chi-square analysis. *P*-values < 0.05 were considered significant. *P*-value*: the analysis was adjusted for age, energy intake, physical activity, sex, and BMI.

### Characteristics of participants according to nitrite from animal and plant sources

Table 4 shows the characteristics of participants based on high/low nitrite intake and its dietary sources (animal or plant). Participants with higher nitrite intake (≥12.15 mg/day) had lower BMI, LDL, and SBP. While participants with higher nitrite intake from animal sources (≥6.27 mg/day) had higher weight, BMI, WC, FBS, AST, and SBP, Participants with higher nitrite intake from plant sources (≥5.62 mg/day) had lower TG, ALT, AST, and SBP.

**Table 4:** Dietary intakes of participants among nitrate and nitrite intake.

Variables	Nitrate (mg/day)		*P* value	*P* value *	Nitrite (mg/day)		*P* value	*P* value *
	Low intake (n = 115)	High intake (n = 135)			Low intake (n = 113)	High intake (n = 137)		
Macronutrient								
Energy (kcal/day)	2337.30 (541.04)	3103.77 (587.68)	**<0.001**	**<0.001**	2303.33 (517.60)	3146.97 (554.37)	**<0.001**	**<0.001**
Cho (gr/day)	330.28 (85.65)	442.27 (95.67)	**<0.001**	**0.02**	337.50 (92.26)	433.10 (97.79)	**<0.001**	**<0.001**
Fat (gr/day)	76.60 (23.53)	100.64 (27.21)	**<0.001**	**0.03**	71.82 (18.94)	106.72 (25.10)	**<0.001**	**<0.001**
Protein (gr/day)	88.13 (23.21)	118.19 (25.87)	**<0.001**	**<0.001**	83.51 (17.99)	124.07 (22.96)	**<0.001**	**<0.001**
Micronutrient								
Minerals								
Ca (mg/day)	828.85 (299.65)	1135.64 (362.07)	**<0.001**	**<0.001**	819.58 (276.07)	1147.43 (375.34)	**<0.001**	**<0.001**
P (mg/day)	997.16 (348.22)	1379.91 (402.49)	**<0.001**	**<0.001**	941.27 (292.70)	1450.99 (379.17)	**<0.001**	**<0.001**
Mg (mg/day)	196.70 (56.03)	283.42 (74.50)	**<0.001**	**<0.001**	190.43 (48.83)	291.38 (70.82)	**<0.001**	**<0.001**
Fe (mg/day)	17.16 (4.57)	23.30 (5.28)	**<0.001**	**<0.001**	17.14 (4.69)	23.32 (5.12)	**<0.001**	0.75
Zinc (mg/day)	6.60 (2.09)	9.04 (2.50)	**<0.001**	**<0.001**	6.20 (1.68)	9.55 (2.31)	**<0.001**	**<0.001**
Copper (mg/day)	1.01 (0.44)	1.45 (0.82)	**<0.001**	**<0.001**	0.95 (0.39)	1.52 (0.81)	**<0.001**	**<0.001**
Na (mg/day)	2817.13 (746.84)	3651.48 (975.06)	**<0.001**	**0.04**	2652.19 (616.40)	3861.25 (864.86)	**<0.001**	**<0.001**
K (mg/day)	2391.28 (678.35)	3709.37 (1073.76)	**<0.001**	**<0.001**	2355.57 (624.23)	3754.78 (1057.89)	**<0.001**	**<0.001**
Vitamins								
A (IU/day)	1220.30 (798.90)	2497.11 (2065.67)	**<0.001**	**<0.001**	1257.50 (867.10)	2449.81 (2058.97)	**<0.001**	**<0.001**
D (μg/day)	1.20 (1.02)	1.59 (1.29)	**<0.001**	**0.01**	1.15 (0.97)	1.66 (1.32)	**<0.001**	**<0.001**
E (mg/day)	2.64 (0.74)	3.80 (1.14)	**<0.001**	**<0.001**	2.62 (0.73)	3.82 (1.12)	**<0.001**	**<0.001**
K (mg/day)	120.11 (50.03)	193.16 (81.76)	**<0.001**	**<0.001**	125.81 (53.14)	185.91 (85.10)	**<0.001**	**<0.001**
C (mg/day)	88.39 (31.47)	153.74 (67.01)	**<0.001**	**<0.001**	92.53 (34.85)	148.48 (69.61)	**<0.001**	**<0.001**
B1 (mg/day)	1.90 (0.48)	2.51 (0.53)	**<0.001**	0.18	1.94 (0.50)	2.47 (0.54)	**<0.001**	**<0.001**
B2 (mg/day)	1.44 (0.51)	2.03 (0.71)	**<0.001**	**<0.001**	1.36 (0.43)	2.13 (0.68)	**<0.001**	**<0.001**
B3 (mg/day)	24.05 (6.30)	32.33 (7.20)	**<0.001**	0.14	23.38 (5.79)	33.18 (6.66)	**<0.001**	**<0.001**
B6 (mg/day)	1.13 (0.46)	1.70 (0.56)	**<0.001**	**<0.001**	1.10 (0.40)	1.75 (0.56)	**<0.001**	**<0.001**
B12 (mg/day)	3.22 (2.50)	4.97 (8.63)	**<0.001**	**0.03**	2.87 (2.20)	5.41 (8.63)	**<0.001**	**<0.001**
B9 (μg/day)	230.90 (67.62)	357.31 (103.07)	**<0.001**	**<0.001**	234.08 (69.19)	353.26 (106.52)	**<0.001**	**<0.001**
Other								
Fiber (gr/day)	14.52 (3.61)	21.96 (5.79)	**<0.001**	**<0.001**	14.86 (3.80)	21.52 (6.14)	**<0.001**	**<0.001**

All data are presented as mean (±SD). *P*-values < 0.05 were considered significant. *P*-value*: the analysis was adjusted for age, energy intake, physical activity, sex, and BMI.

### Dietary intakes of participants according to nitrate and nitrite intake

Table 5 shows participants’ dietary intakes based on low and high nitrate and nitrite intake. Individuals with higher nitrate and nitrite intakes had higher macro and micronutrient intake.

**Table 5:** Interaction between nitrate and nitrite intake from animal and plant sources and TMAO levels on metabolic syndrome and its components.

Variables	Models	TMAO levels (pg/ml)	Nitrate (mg/day)												Nitrite (mg/day)											
			Total				Animal source				Plant source				Total				Animal source				Plant source			
			High adherence				High adherence				High adherence				High adherence				High adherence				High adherence			
			β	OR	95%CI	P value	β	OR	95%CI	P value	β	OR	95%CI	P value	β	OR	95%CI	P value	β	OR	95%CI	P value	β	OR	95%CI	P value
MetS+	Crude	Low	Reference																							
		High	−0.14	0.86	0.27–2.70	0.79	−0.04	0.95	0.30–2.99	0.93	−0.52	0.59	0.18–1.86	0.37	−0.12	0.88	0.28–2.79	0.83	0.05	1.06	0.33–2.37	0.92	−0.89	0.40	0.12–1.28	**0.09**
	Adjusted	Low	Reference																							
		High	−0.08	0.91	0.28–2.96	0.88	0.04	1.04	0.32–3.37	0.94	−0.47	0.64	0.23–1.93	0.36	−0.10	0.89	0.27–2.95	0.86	0.03	1.23	0.31–2.43	0.95	−0.80	0.44	0.13–1.46	**0.06**
Hypertriglyceridemia	Crude	Low	Refrence																							
		High	−0.68	0.50	0.16–1.57	0.23	0.31	1.35	0.30–2.91	**0.09**	−0.57	0.56	0.18–1.76	0.22	−0.35	0.69	0.22–2.18	0.56	−0.26	0.76	0.24–2.44	0.34	−1.41	0.24	0.07–0.76	**0.01**
	Adjusted	Low	Reference																							
		High	−0.66	0.55	0.14–1.63	0.25	0.30	1.36	0.34–1.87	**0.07**	−0.53	0.58	0.18–1.85	**0.05**	−034	0.71	0.35–2.28	0.53	0.45	1.57	0.35–2.87	**0.05**	−1.33	0.26	0.08–0.84	**0.02**
Hyper FBS	Crude	Low	Reference																							
		High	−0.80	0.44	0.10–1.89	0.27	−0.70	0.49	0.12–1.97	0.31	−0.74	0.47	0.11–1.94	0.27	−0.38	0.67	0.17–2.67	0.58	0.03	1.03	0.26–2.03	0.95	−1.48	0.22	0.05–1.02	**0.05**
	Adjusted	Low	Reference																							
		High	−0.85	0.42	0.09–1.82	0.25	−0.82	0.44	0.10–1.85	0.26	−0.79	0.45	0.10–1.87	**0.04**	−0.45	0.63	0.15–2.58	0.52	−0.08	0.91	0.22–2.71	0.90	−1.52	0.21	0.04–0.94	**0.05**
Hypertension	Crude	Low	Reference																							
		High	−0.05	0.95	0.21–2.12	0.94	0.18	1.20	0.27–2.23	0.80	−0.08	0.92	0.20–2.94	0.27	0.47	1.61	0.35–2.28	0.53	0.45	1.57	0.35–2.87	0.39	−0.23	0.79	0.18–2.51	0.26
	Adjusted	Low	Reference																							
		High	−0.14	0.86	0.19–2.90	0.85	0.15	1.15	0.26–2.14	0.73	−0.11	0.89	0.26–2.49	**0.07**	0.46	1.59	0.34–2.42	0.54	0.42	1.53	0.33–2.88	**0.05**	−0.30	0.74	0.16–2.31	**0.06**
Abdominal obesity	Crude	Low	Reference																							
		High	−0.05	0.95	0.30–2.95	0.93	0.23	1.26	0.40–2.91	0.32	0.08	1.08	0.35–2.36	0.38	−0.07	0.93	0.29–2.95	0.90	−0.51	0.60	0.19–1.88	0.38	−0.26	0.76	0.24–2.36	0.83
	Adjusted	Low	Reference																							
		High	0.18	1.20	0.33–2.35	0.78	0.59	1.80	0.49–2.58	**0.08**	0.21	1.23	0.34–2.46	0.44	−0.18	0.83	0.22–2.07	0.78	−0.58	0.55	0.15–2.04	0.37	−0.22	0.70	0.27–2.59	0.64
Low HDL	Crude	Low	Reference																							
		High	0.06	1.06	0.28–2.90	0.92	0.33	1.39	0.38–2.08	0.61	−0.04	0.95	0.26–2.49	0.74	0.69	2.01	0.52–2.67	0.30	0.70	2.02	0.53–2.72	0.30	−0.01	0.98	0.27–2.61	0.88
	Adjusted	Low	Reference																							
		High	0.34	1.40	0.32–2.13	0.64	0.30	1.36	0.31–2.91	0.68	0.25	1.29	0.29–2.58	0.94	0.60	1.82	0.40–2.26	0.43	0.67	1.96	0.42–2.03	**0.04**	0.20	1.22	0.28–2.78	0.78

TMAO: trimethylamine N-Oxide, MetS: metabolic syndrome, FBS: fasting blood sugar, HDL: high-density lipoprotein. Plant sources include: grains, vegetables, legumes, and nuts. Animal sources include: meat, poultry, processed meats, and dairy products. *P*-values < 0.10 were considered significant. *P*-value*: the analysis was adjusted for age, energy intake, BMI, physical activity, smoking, sex, education, marriage status, SES, use of multivitamins, and intake of vitamin C. TMAO < 30.39 pg/mL, low adherence total nitrate (631.25 mg/day), low adherence animal nitrate (22.28 mg/day), low adherence plant nitrate (595.18 mg/day), low adherence total nitrite (12.15 mg/day), low adherence animal nitrate (6.27 mg/day), low adherence plant nitrate (5.62 mg/day), MetS-, normal values of TG, FBS, WC, BP, and HDL were considered the reference group.

### Interaction between nitrate and nitrite from animal and plant sources and TMAO on MetS

**Figure 1:**
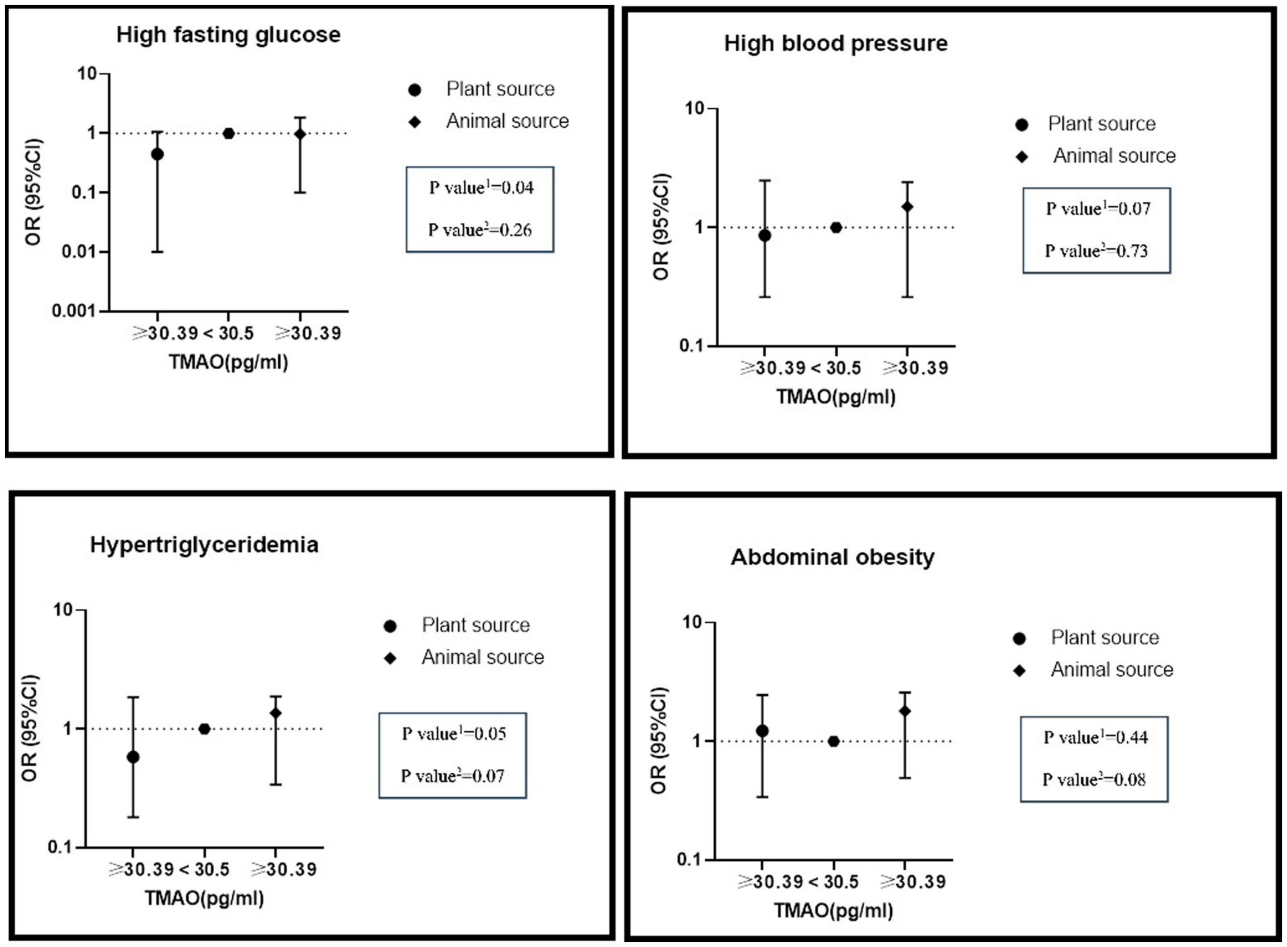
The interaction of dietary nitrate intake from plant and animal sources with TMAO metabolite on metabolic syndrome and its components in the modified model.

The interaction between high levels of TMAO (>30.39 pg./mL) and higher intake of nitrate/nitrite from animal and plant sources on MetS and its components is presented in Table 6. In adjusted models, there was a significant positive interaction between high TMAO levels and the highest nitrate intake from animal sources on hypertriglyceridemia (OR: 1.36, %95CI: 0.34–1.87, P: 0.07) and abdominal obesity (OR: 1.80, %95CI: 0.49–2.58, P: 0.08). Furthermore, there was a significant negative interaction between high TMAO levels and the highest nitrate intake from plant sources on hypertriglyceridemia (OR: 0.58, %95CI: 0.18–1.85, P: 0.05), hyper FBS (OR: 0.45, %95CI: 0.10–1.87, P: 0.04) and hypertension (OR: 0.89, %95CI: 0.26–2.49, P: 0.07) (Figure 1).

**Table 6:** Interaction between nitrate and nitrite intake from animal and plant sources and KYN levels on Metabolic syndrome and its components.

Variables	Models	KYN levels (nmol/l)	Nitrate (mg/day)												Nitrite (mg/day)											
			Total				Animal source				Plant source				Total				Animal source				Plant source			
			High adherence				High adherence				High adherence				High adherence				High adherence				High adherence			
			β	OR	95%CI	*P* value	β	OR	95%CI	*P* value	β	OR	95%CI	*P* value	β	OR	95%CI	*P* value	β	OR	95%CI	P value	β	OR	95%CI	P value
Mets+	Crude	Low	Reference																							
		High	−0.68	0.50	0.16–1.55	0.23	0.33	0.71	0.23–2.20	0.56	−0.86	0.41	0.13–1.29	0.26	−0.04	0.67	0.21-2.07	0.48	0.55	1.74	0.56–2.38	0.33	−0.37	0.68	0.22–2.09	0.50
	Adjusted	Low	Reference																							
		High	−47	0.62	0.19–2.01	0.43	−0.36	0.69	0.21–2.25	0.54	−0.66	0.51	0.16–1.65	**0.08**	−0.51	0.59	0.18–1.94	0.39	0.59	1.81	0.55–2.86	0.30	−0.23	0.79	0.24–2.54	0.69
Hypertriglyceridemia	Crude	Low	Refrence																							
		High	−0.70	0.49	0.16–1.51	0.21	0.32	1.26	0.26–2.70	**0.02**	−0.85	0.43	0.13–1.29	0.13	−1.02	0.35	0.11–1.11	**0.07**	−0.46	0.63	0.20–1.92	0.41	−0.69	0.49	0.16–1.51	0.33
	Adjusted	Low	Reference																							
		High	−0.53	0.58	0.18–1.84	0.36	0.33	1.29	0.30–2.42	**0.01**	−0.69	0.50	0.15–1.57	0.23	−1.06	0.34	0.10–1.10	**0.06**	−0.43	0.64	0.20–2.03	0.45	−0.56	0.56	0.18–1.78	**0.08**
Hyper FBS	Crude	Low	Reference																							
		High	−1.58	0.20	0.04–0.92	**0.03**	−0.34	0.70	0.17–2.83	0.62	−2.01	0.13	0.03–0.60	**0.009**	−0.31	0.73	0.18–2.92	0.66	0.44	1.55	0.39–2.14	0.52	−1.48	0.22	0.05–1.01	**0.01**
	Adjusted	Low	Reference																							
		High	−1.72	0.17	0.03–0.82	**0.02**	−0.37	0.68	0.16–2.88	0.60	−2.20	0.11	0.02–0.51	**0.005**	−0.26	0.76	0.18–2.16	0.71	0.48	1.61	0.37–2.06	0.50	−1.52	0.21	0.04–0.99	**0.04**
Hypertension	Crude	Low	Reference																							
		High	−0.57	0.56	0.13–2.44	0.44	−0.30	0.73	0.17–2.16	0.67	−0.57	0.56	0.12–2.46	0.44	−0.66	0.51	0.11–2.38	0.39	1.18	2.25	1.73–3.84	0.12	−0.76	0.46	0.10–2.03	0.24
	Adjusted	Low	Reference																							
		High	−0.80	0.44	0.09–2.02	0.29	−0.53	0.58	0.13–2.62	0.48	−0.70	0.49	0.10–2.27	0.36	−0.71	0.48	0.10–2.34	0.37	1.15	2.17	1.69–3.40	**0.05**	−0.90	0.40	0.09–1.83	**0.07**
Abdominal obesity	Crude	Low	Reference																							
		High	−0.54	0.57	0.18–1.77	0.34	−0.05	0.94	0.30–2.91	0.91	−0.01	0.98	0.26–2.59	0.97	−0.13	0.87	0.27–2.74	0.81	−0.09	0.90	0.29–2.77	0.86	−0.39	0.67	0.21–2.12	0.50
	Adjusted	Low	Reference																							
		High	−0.44	0.63	0.20–1.98	0.54	−0.02	0.97	0.26–2.65	0.87	−0.58	0.55	0.18–1.70	0.30	−0.08	0.92	0.24–2.45	0.90	0.33	1.39	0.37–2.19	0.61	−0.67	0.50	0.13–1.92	0.31
Low HDL	Crude	Low	Reference																							
		High	0.07	1.07	0.31–2.72	0.80	1.17	2.24	1.91–3.52	**0.05**	0.07	1.07	0.31–2.74	0.48	0.39	1.42	0.39–2.20	0.42	1.25	2.33	1.82–3.65	**0.05**	0.18	1.20	0.34–2.16	0.77
	Adjusted	Low	Reference																							
		High	0.60	1.83	0.42–2.84	0.41	1.22	2.39	1.78–3.87	**0.01**	0.63	1.88	0.43–2.10	0.89	0.45	1.58	0.40–2.64	0.38	1.38	2.44	1.92–3.89	**0.07**	0.36	1.43	0.38–2.96	0.62

KYN: kynurenine, MetS: metabolic syndrome, FBS: fasting blood sugar, HDL: high-density lipoprotein. Plant sources include: grains, vegetables, legumes, and nuts. Animal sources include: meat, poultry, processed meats, and dairy products. *P*-values < 0.10 were considered significant. *P*-value*: the analysis was adjusted for age, energy intake, BMI, physical activity, smoking, sex, education, marriage status, SES, use of multivitamins, and intake of vitamin C. KYN < 297.18 nmoL/L, low adherence total nitrate (631.25 mg/day), low adherence animal nitrate (22.28 mg/day), low adherence plant nitrate (595.18 mg/day), low adherence total nitrite (12.15 mg/day), low adherence animal nitrate (6.27 mg/day), low adherence plant nitrate (5.62 mg/day), MetS-, normal values of TG, FBS, WC, BP and HDL were considered the reference group.

**Figure 2:**
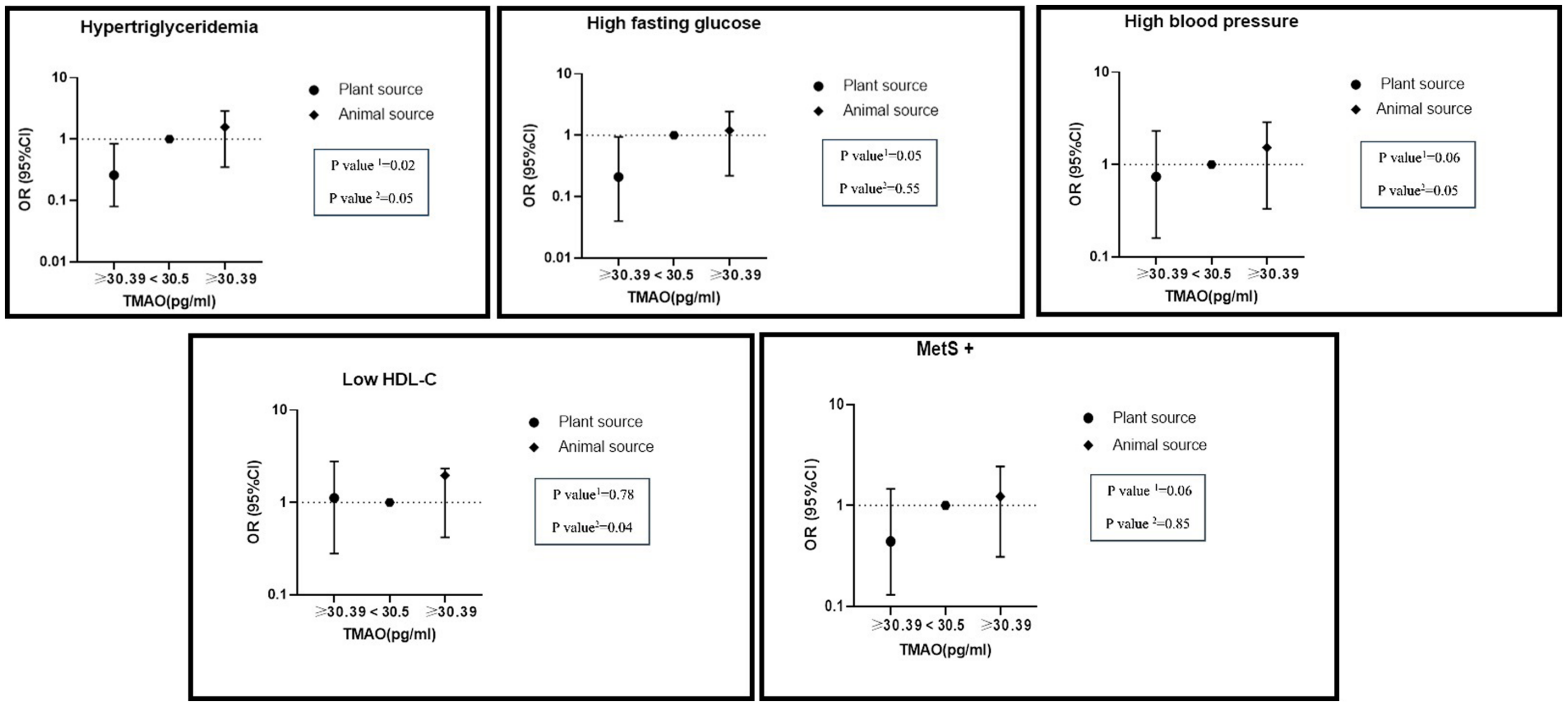
Interaction of dietary nitrite intake form plant and animal sources with TMAO metabolite on metabolic syndrome and its components in adjusted model. Number 1 represents plant resources and number 2 animal resources.

Additionally, a significant interaction between high TMAO levels and high intake of nitrite from animal sources on hypertriglyceridemia (OR: 1.57, %95CI: 0.35–2.87, P: 0.05), hypertension (OR: 1.53, %95CI: 0.33–2.58, P: 0.06) and reduced HDL (OR: 1.96, %95CI: 0.42–2.03, P: 0.04) was found in the adjusted model. High consumption of nitrite from plant sources with high levels of TMAO had lower odds of MetS (OR: 0.44, %95CI: 0.13–1.46, P: 0.06), hypertriglyceridemia (OR: 0.26, %95CI: 0.08–0.84, P: 0.02), hyper FBS (OR: 0.21, %95CI: 0.04–0.94, P: 0.05) and hypertension (OR: 0.74, %95CI: 0.16–2.31, P: 0.09) (Figure 2).

### Interaction between nitrate and nitrite intake from animal and plant sources and KYN levels on MetS

The interaction between the intake of nitrate and nitrite from animal and plant sources and KYN levels on MetS and its components is shown in Table 6.

**Figure 3:**
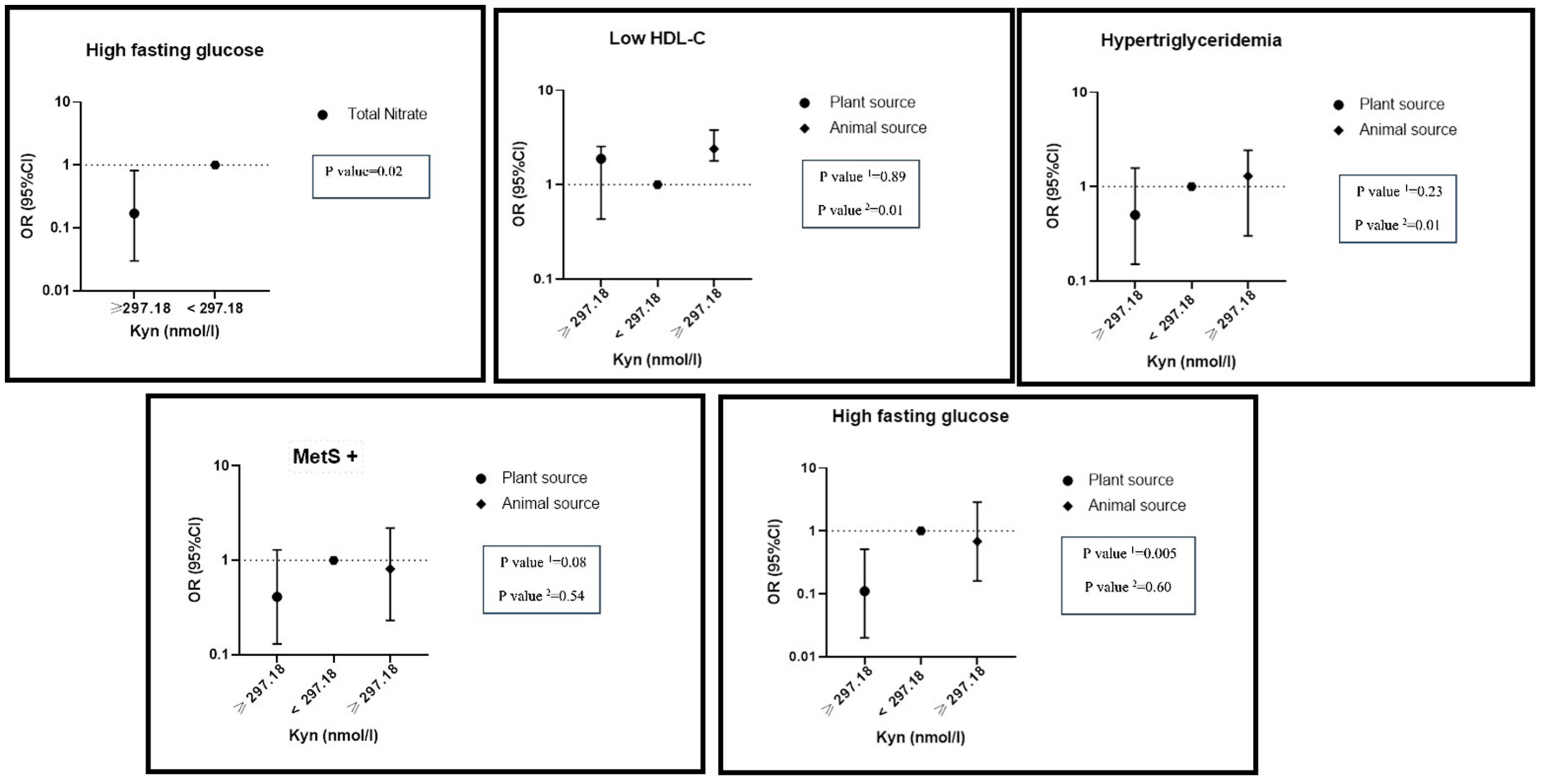
Interaction of dietary nitrate intake form plant and animal sources with KYN metabolite on metabolic syndrome and its components in adjusted model. Number 1 represents plant resources and number 2 animal resources.

There was a significant negative interaction between total nitrate intake and a high level of KYN on hyperFBS (OR: 0.17, %95CI: 0.03–0.82, P: 0.02). While there was a significant interaction between high levels of KYN and high intake of nitrate from animal sources on hypertriglyceridemia (OR: 1.29, %95CI: 0.30–2.42, P: 0.01) and lower HDL (OR: 2.39, %95CI: 1.78–3.87, P: 0.01) levels, a significant negative interaction between high levels of KYN and high nitrate intake from plant sources on MetS (OR: 0.51, %95CI: 0.16–1.65, P: 0.08) and hyper FBS (OR: 0.11, %95CI: 0.02–0.51, P: 0.005) was observed (Figure 3).

**Figure 4:**
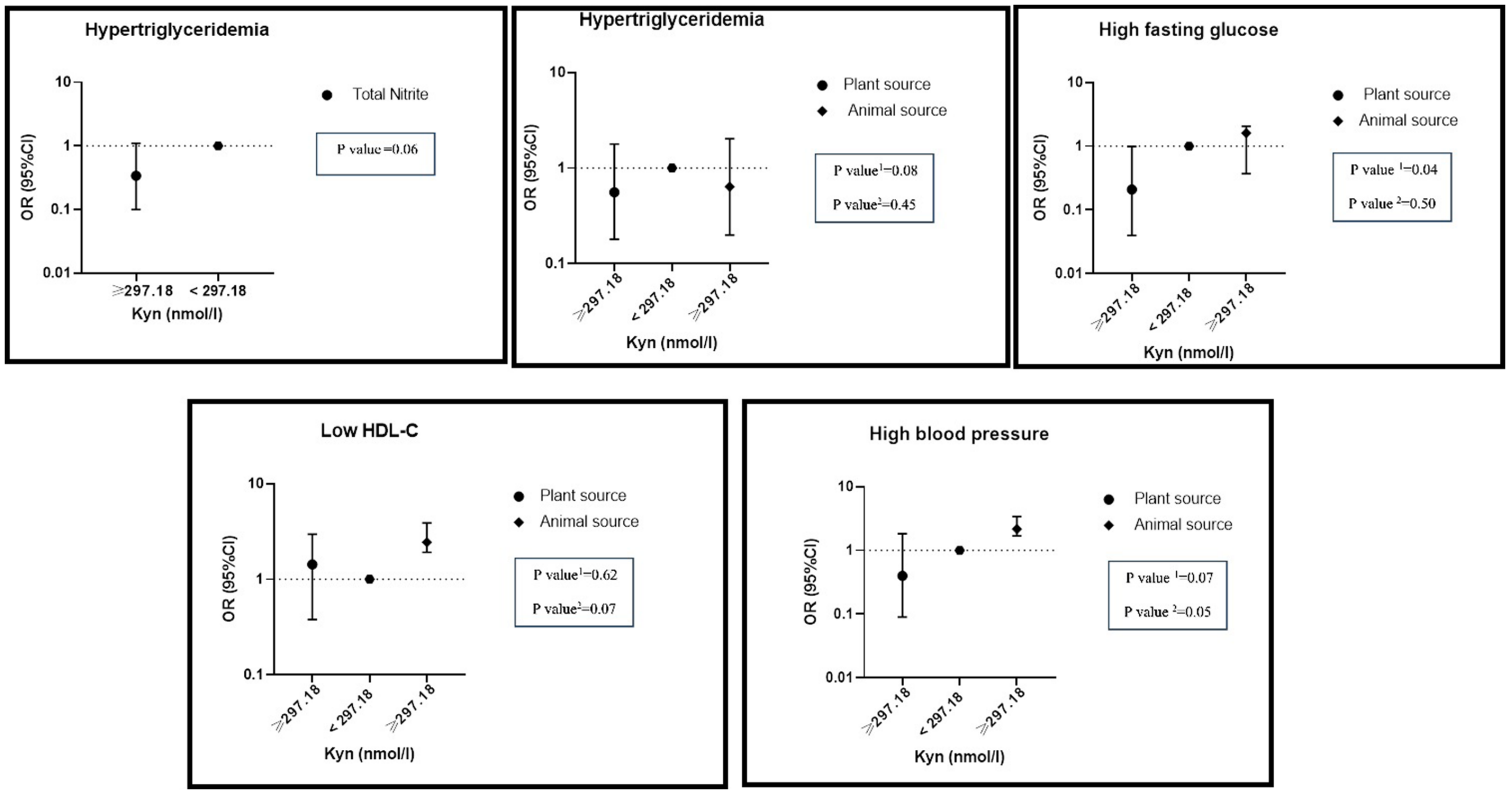
Interaction of dietary nitrite intake form plant and animal sources with KYN metabolite on metabolic syndrome and its components in adjusted model. Number 1 represents plant resources and number 2 animal resources.

There was a significant interaction between high levels of KYN and the highest adherence to total nitrite intake on hypertriglyceridemia (OR: 0.34, %95CI: 0.10–1.10, P: 0.06). A significant interaction between high levels of KYN and the highest adherence to nitrite intake from animal sources was found on reduced HDL (OR: 2.44, %95CI: 1.92–3.89, P: 0.07) and hypertension (OR: 2.17, %95CI: 1.69–3.40, P: 0.05). On the other hand, there was a negative interaction between the highest nitrite intake from plant sources and high levels of KYN on hypertension (OR: 0.40, %95CI: 0.09–1.83, P: 0.07), hypertriglyceridemia (OR: 0.56, %95CI: 0.18–1.78, P: 0.08) and hyper FBS (OR: 0.21, %95CI: 0.04–0.99, P: 0.04) (Figure 4).

## Discussion

**Figure 5:**
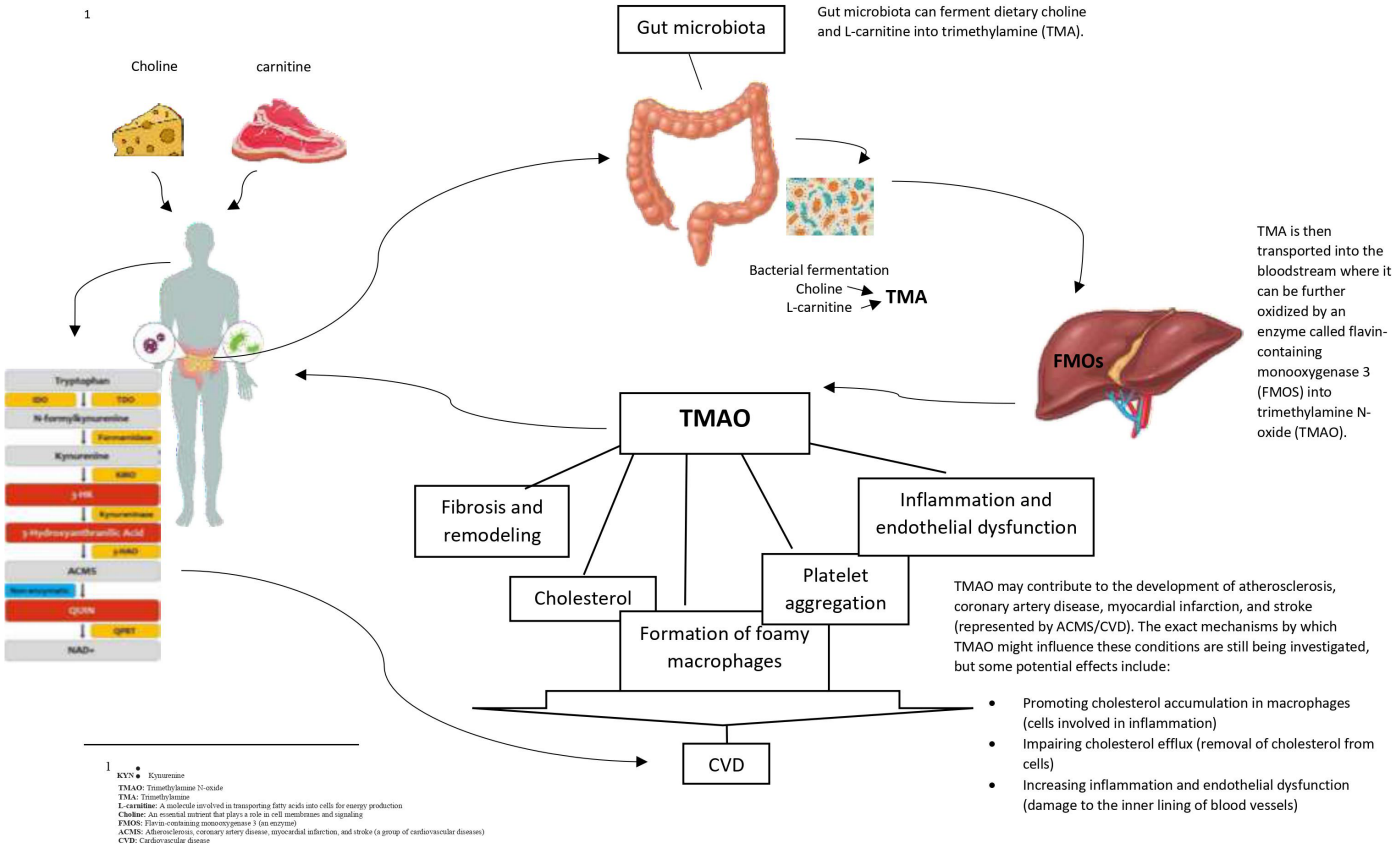
Mechanism of generated TMAO and effect of organs CVD.

To the best of our knowledge, this study for the first time, investigated the interaction between nitrate and nitrite intake and TMAO and KYN levels on MetS and its components. There was a negative interaction between total nitrate intake and high levels of KYN on hyperFBS and between high KYN levels and total nitrite intake on hypertriglyceridemia. Furthermore, a significant positive interaction between high TMAO and KYN levels, and high nitrate and nitrite intake from animal sources on some components of MetS was found, while a significant negative interaction between high TMAO and KYN levels and nitrate and nitrite intake from plant sources on some components of MetS was observed (see Figure 5).

A significant positive association between higher levels of TMAO and increased WHR, WC, and HC was found. Furthermore, the elevated levels of FBS, liver enzymes, and TG were associated with higher levels of TMAO. Consistent with these findings, a meta-analysis demonstrated a positive correlation between TMAO levels and obesity (49). The mechanisms underlying the association between TMAO and obesity are not fully understood. Flavin-containing monooxygenase 3 (FMO3), the enzyme responsible for TMAO production is involved in obesity and the beginning of white adipose tissue (23, 24). In line with our finding on the association between TMAO and TG and FBS, a meta-analysis found a positive dose-dependent association between TMAO levels and increased CVD risk and mortality (50). In addition, in clinical studies, higher plasma levels of TMAO were associated with an increased risk of atherosclerotic cardiovascular disease and total mortality (50–54). However, the current evidence is mixed. A study on North American adults found no association between TMAO levels and incident coronary artery calcification (55). Similarly, another study including 339 patients who underwent coronary angiography showed no association between plasma TMAO levels and incident cardiovascular events (56). The conflicting evidence on the association between TMAO and CVD risk could be attributed to various study methodologies, diet, ethnicity, and gut microbiome composition in participants. The association between TMAO and CVD risk may be due to the fact that TMAO develops cholesterol accumulation in macrophages by upregulating the cell surface expression of proatherogenic scavenger receptors CD36 and SR-A1. This accumulation inhibits reverse cholesterol transport and alters sterol metabolism (57–59). However, the specific animal sources of TAMO were not examined in this study. Given seafood contains high TAMO levels and considered protective for CVD, further studies are needed to examine associations between TAMO from seafood and CVD risk factors (60).

A significant positive association between KYN levels and increased WHR, WC, HC, FBS, ALK, ALT, and AST was found. The evidence shows that the KYN pathway, downstream from TRP, plays a role in obesity, CVD risk factors, inflammation, and insulin resistance (61, 62). Previous studies reported that the increased levels of TC, ALK, FBS, and liver enzymes were associated with higher levels of KYN (63, 64). It is widely recognized that obesity and inflammatory conditions are linked to a shift in the KYN pathway, which results in elevated levels of serum KYN (65–67). Consistent with our findings, a cohort study reported a positive correlation between kynurenine levels and BMI and a higher HOMA-IR (66). The involvement of the tryptophan-kynurenine pathway in the pathophysiology of T2D is yet fully established. However, the evidence suggests a potential link between inflammatory processes and the TRP/KYN pathway activation in T2D (68).

Our results indicated that participants with a higher total nitrate and nitrite intake had lower BMI, FBS, ALT, AST, LDL, and SBP. Furthermore, the findings showed a protective impact of higher total nitrate consumption from plant sources on obesity and several blood parameters and blood pressure levels. On the contrary, nitrate intake from animal sources negatively affected blood parameters and blood pressure. Studies conducted on rats with T2D demonstrated that consuming water enriched with nitrate improved fasting glucose, insulin resistance, insulin sensitivity, and lipid profiles (69, 70). Clinical studies reported that dietary nitrates can improve endothelial dysfunction and vascular stiffness in older adult individuals (71). Furthermore, a systematic review found that acute intake of inorganic nitrate significantly lowered resting blood pressure, and improved endothelial function (72).

There was a negative interaction between total nitrate and nitrite intake and high levels of KYN on hyperFBS and hypertriglyceridemia. There is very limited evidence on the interaction between nitrate and nitrite intake and gut microbial metabolites and MetS components. A study on mice found that nitrate intake improved left ventricle reactive oxygen species, adipose inflammation, lipid homeostasis, glucose intolerance, and gut dysbiosis (19). Furthermore, a study on 9,180 American adults found that higher fiber intake was linked to the production of gut microbial indole propionate, which is associated with a reduced risk of T2DM (73). The mechanism of this effect might be attributed to NO production. A decrease in the availability and/or bioactivity of NO has been associated with the development of hypertension, endothelial dysfunction, atherosclerosis, and CVD (74, 75). The evidence suggests that dietary nitrate intake significantly increases NO availability through the nitrate-nitrite-NO pathway (76–78).

A significant positive interaction between high TMAO and KYN levels, and high nitrate and nitrite intake from animal sources on some components of MetS was found, while opposite findings were observed for nitrate and nitrite intake from plant sources. The beneficial effects from plant sources might be due to vegetable components, such as phenols and ascorbic acid, tocopherols, carotenoids, and flavonoids that prevent the toxic effects of nitrites (79, 80). Furthermore, certain dietary compounds like vitamin C and polyphenols enhance the conversion of nitrites into NO and protect NO from oxidative degradation (81, 82). Two studies reported that plant-based diets with a high intake of vegetable and fruit juices (including beetroot) could increase fecal Bacteroidetes and decrease Firmicutes, a phenotype linked to lower obesity (83, 84).

This study has several strengths. To the best of our knowledge, this is the first study that examined the interaction between two important gut metabolites and the intake of nitrate and nitrite on Mets. Measuring these two factors together provides more reliable evidence than assessing them individually. However, this study has several limitations to be considered in interpreting its findings. Firstly, this study used an FFQ to collect dietary intake, which relies on individuals’ memory. Secondly, the metabolites derived from gut microbiota were only measured in serum, not fecal samples. Thirdly, the sample size of the study is limited due to the limitations in the budget and the measurement methods of the study. Fourthly, there may be further confounding factors including genetic predisposition, medication use, socio-economic status, and other lifestyle habits that may also influence associations found in this study. Lastly, the cross-sectional study design makes it challenging to establish a cause-and-effect relationship.

### Future directions

Given the current mixed findings on associations between TAMO and KYN levels and CVD risk factors, furtherstudies with larger sample sizes evaluating these metabolites in serum and fecal samples are needed to investigate associations between these metabolites and CVD. Furthermore, considering the interaction effects between dietary nitrate and nitrite intake and these two metabolites, prospective and experimental studies are needed to develop our knowledge in this regard.

## Conclusion

In conclusion, High intake of nitrate and nitrite from animal sources were found to have a significant negative interaction with high serum levels of TMAO and KYN on the probability of developing some components ofMetS. On the other hand, high intake of nitrate and nitrite from plant sources were found to have a significant positive interaction on developing some components ofMetS. Higher intake of nitrate and nitrite from plant sources improved cardiovascular disease risk factors in individuals with elevated serum levels of TMAO and KYN metabolites.

## Data Availability

The datasets presented in this study can be found in online repositories. The names of the repository/repositories and accession number(s) can be found in the article/supplementary material.

## Footnotes

^1^NO: colorless toxic gas that is formed by the oxidation of nitrogen.

^2^TRP: An essential plant-derived amino acid that is needed for the *in vivo* biosynthesis of proteins.

## Glossary

ALK: alkaline phosphatase
ALT: alanine aminotransferase
AST: aspartate aminotransferase
ANCOVA: Analysis of Covariance
BMI: body mass index
CVD: cardiovascular diseases
DBP: diastolic blood pressure
eNOS: cell-derived nitric oxide synthase
FBS: fasting blood sugar
FFQ: Food Frequency Questionnaire
FMO: flavin-containing monooxygenase
HOMA-IR: Homeostatic Model Assessment—Insulin Resistance
HC: hip circumference
HDL_C: high-density lipoprotein cholesterol
IQR: interquartile range
LDL_C: low-density lipoprotein cholesterol
KYN: Kynurenine
KP: Kynurenine pathway
IDO: indole 2,3-dioxygenase
IPAQ: International Physical Activity Questionnaire
MetS: metabolic syndrome
NAFLD: Non-alcoholic fatty liver disease
NO: Nitric oxide
NCEP ATPIII: National Cholesterol Education Program Adult Treatment Panel III
PA: Physical activity
RNS: reactive nitrogen species
SBP: systolic blood pressure
SES: social economic status
SD: standard deviation
T2D: type 2 diabetes
TC: total cholesterol
TDO: tryptophan 2,3-dioxygenase
TG: triglycerides
TMA: Trimethylamine
TMAO: Trimethylamine oxide
TRP: tryptophan
WC: waist circumference
WHR: waist-hip ratio
